# Does access to care play a role in liver cancer survival? The ten-year (2006–2015) experience from a population-based cancer registry in Southern Italy

**DOI:** 10.1186/s12885-021-07935-0

**Published:** 2021-03-24

**Authors:** Walter Mazzucco, Francesco Vitale, Sergio Mazzola, Rosalba Amodio, Maurizio Zarcone, Davide Alba, Claudia Marotta, Rosanna Cusimano, Claudia Allemani

**Affiliations:** 1 Department for Health Promotion, Maternal and Infant Care, Internal Medicine and Medical Specialties (PROMISE), University of Palermo, Palermo, Italy; 2 Clinical Epidemiology and Cancer Registry Unit, Palermo University Hospital “P. Giaccone”, Palermo, Italy; 3 Division of Biostatistics and Epidemiology, Cincinnati Children’s Hospital Medical Centre, Cincinnati, OH USA; 4 Department of Paediatrics, University of Cincinnati College of Medicine, Cincinnati, OH USA; 5 Palermo Health Agency, Palermo, Italy; 6 Cancer Survival Group, Department of Non-Communicable Disease Epidemiology, London School of Hygiene & Tropical Medicine, London, UK

**Keywords:** liver cancer, access to care, survival, cancer registries, public health

## Abstract

**Background:**

Hepatocellular carcinoma (HCC) is the most frequent primary invasive cancer of the liver. During the last decade, the epidemiology of HCC has been continuously changing in developed countries, due to more effective primary prevention and to successful treatment of virus-related liver diseases.

The study aims to examine survival by level of access to care in patients with HCC, for all patients combined and by age.

**Methods:**

We included 2018 adult patients (15–99 years) diagnosed with a primary liver tumour, registered in the Palermo Province Cancer Registry during 2006–2015, and followed-up to 30 October 2019. We obtained a proxy measure of access to care by linking each record to the Hospital Discharge Records and the Ambulatory Discharge Records.

We estimated net survival up to 5 years after diagnosis by access to care (“easy access to care” versus “poor access to care”), using the Pohar-Perme estimator. Estimates were age-standardised using International Cancer Survival Standard (ICSS) weights. We also examined survival by access to care and age (15–64, 65–74 and ≥ 75 years).

**Results:**

Among the 2018 patients, 62.4% were morphologically verified and 37.6% clinically diagnosed. Morphologically verified tumours were more frequent in patients aged 65–74 years (41.6%), while tumours diagnosed clinically were more frequent in patients aged 75 years or over (50.2%). During 2006–2015, age-standardised net survival was higher among HCC patients with “easy access to care” than in those with “poor access to care” (68% vs. 48% at 1 year, 29% vs. 11% at 5 years; *p* < 0.0001). Net survival up to 5 years was higher for patients with “easy access to care” in each age group (*p* < 0.0001). Moreover, survival increased slightly for patients with easier access to care, while it remained relatively stable for patients with poor access to care.

**Conclusions:**

During 2006–2015, 5-year survival was higher for HCC patients with easier access to care, probably reflecting progressive improvement in the effectiveness of health care services offered to these patients.

Our linkage algorithm could provide valuable evidence to support healthcare decision-making in the context of the evolving epidemiology of hepatocellular carcinoma.

## Background

Despite important diagnostic and therapeutic advances, liver cancer remains highly lethal in both developing and developed countries [1, 2]. Liver cancer is the fifth most common cancer world-wide in men, the seventh in women, and it is the second most common cause of cancer death, with an estimated 782,000 new cases and 746,000 deaths per year [3].

Hepatocellular carcinoma (HCC) is the most frequent primary invasive cancer of the liver, accounting for 60–80% of all invasive malignancies of the liver [4, 5]. Its development is closely related to the presence of chronic liver disease with hepatitis B or C [6]. During the last decade, the epidemiology of HCC has been continuously changing in developed countries, due to more effective primary prevention and to a successful treatment of virus-related liver diseases, which highlighted the impact of emerging risk factors, other than the well-documented risk from excess alcohol consumption [7], and a consequent shift to non-alcoholic fatty liver disease [8].

Population-based cancer registries have a quintessential role in generating real-world evidence on cancer incidence, survival and the quality of cancer care [9–11]. Furthermore, registries are also challenged to develop methods that can produce evidence on the real-world impact of care pathways in support of healthcare decision-makers [12, 13].

Population-based cancer survival trends provide researchers and policy makers with evidence of the effectiveness of control programmes, reflecting access to healthcare, early diagnosis and optimal treatment [14]. The third cycle of the CONCORD programme for global surveillance of population-based cancer survival trends documented very little improvement in age-standardised 5-year net survival for liver cancer between 1995 and 1999 and 2010–2014, in 61 countries. Five-year survival was generally in the range 3–30% [1].

A more detailed analysis on patients diagnosed during 1995–2009 in 28 countries found a wide variation in 5-year conditional survival (the probability of surviving up to 5 years from diagnosis among patients who had survived to the first anniversary of diagnosis) for hepatocellular carcinomas (25–52%). This partially reflects variation in the proportion of patients diagnosed at an advanced stage [15] whereas a poor prognosis for liver cancer implies that most patients are diagnosed when they are inoperable.

In 2010–2014, age-standardised 5-year net survival for liver cancer was 20.3% (95%CI: 19.6–21.1) in Italy, among the highest in the world, together with Korea, Singapore, Taiwan and Belgium [1]. Moreover, about 90% of liver cancer in southern Italy is estimated to be attributable to infection with hepatitis B virus (HBV) or hepatitis C virus (HCV) [16]. Of interest, during the last 10 years in Italy, an improvement in HCC survival was observed [17]. This is likely to be due both to improvement in clinical surveillance and to the availability of innovative drugs [5], thus leading to an evolution of the aetiology and the epidemiological scenario of liver cancer, related to a progressive reduction of viral aetiology and a consequent increase in the proportion of cases due to non-viral causes [17].

Identification of pathways undertaken by patients diagnosed with cancer is an important issue to improve access to care and, consequently, health outcomes such as survival. Patients with poor access to health facilities have lower survival, than those with easier access [18].

This study aims to investigate primary HCC survival up to 5 years since diagnosis and to highlight any difference in survival by the level of access to care.

For this purpose, we analysed data on patients with liver cancer, collected by the Palermo Province Cancer Registry (PPCR), during 10 years of epidemiological surveillance in an area with a population of 1,276,525 inhabitants [19], characterised by the presence of 3 highly specialised clinical centres for HCV treatment. We applied a deterministic algorithm linkage to both morphologically confirmed tumours and cancer diagnosed on the basis of clinical investigation only, using all available healthcare information sources.

## Methods

We included 2018 adults (15–99 years) among residents in the Palermo Province, who were diagnosed during 2006–2015 with a primary invasive liver neoplasm (C22.0, behaviour code 3, in the International Classification of Diseases for Oncology - ICD-O-3, Third Edition) [20], registered in the Palermo Province Cancer Registry. We excluded tumours arising from intrahepatic biliary ducts (C22.1). We excluded from survival analysis 174 (8.6%) patients who were diagnosed only from a death certificate (DCO). We divided liver cancers into two subgroups on the basis of morphology code [20] and according to European Network of Cancer Registries (ENCR) recommendations [21]: 1) malignant neoplasms with a defined morphology, namely epithelial liver tumours (ICD-O-3 codes 8170–8175; 8010; 8020; 8021; 8190; 8246; 8249); 2) malignant neoplasms with a clinical-instrumental diagnosis (morphology code 8000, behaviour code 3).

### Linkage algorithm

**Fig. 1:**
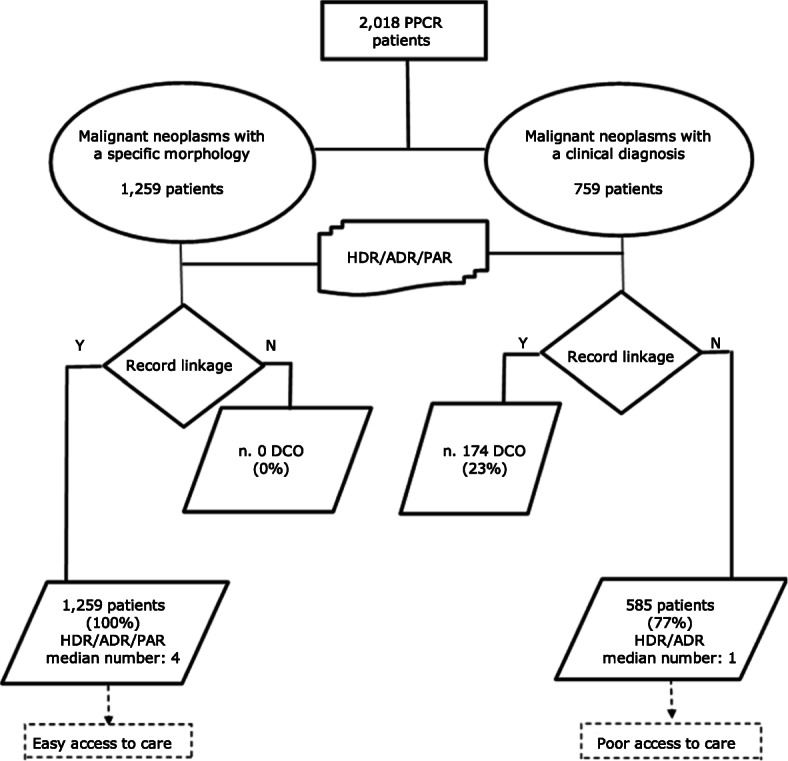
Access to care linkage algorithm for patients with primary invasive liver neoplasms. Palermo Province Cancer Registry, 2006–2015. PPCR = Palermo Province Cancer Registry; HDR = Hospital Discharge Records; ADR = Ambulatory Discharge Records; PAR = Pathological Anatomy Report; DCO = Death Certificate Only; Y = yes, linked; N = not linked

We obtained a proxy measure of access to care through the linkage algorithm showed in Fig. 1. We linked 2018 records to the Hospital Discharge Records (HDR), both for ordinary and day-hospital admissions, to the Ambulatory Discharge Records (ADR) and the Pathological Anatomy Reports (PAR). We performed a deterministic linkage based on the tax code for every inpatient and outpatient diagnosed with a primary invasive liver cancer. Assuming that a higher or lower number of contacts with the healthcare service could be considered a proxy for an easier or poorer access to healthcare, we explored access to care, taking into account information derived from different data flows: year of diagnosis, age at diagnosis, and number of HDR, ADR or PAR for every single year of survival after diagnosis. More in depth, we divided patients into two groups: patients linking with at least two different healthcare sources, including PAR (“easy access to care”), and patients linking with only an HDR or ADR, but no PAR (“poor access to care”). Patients diagnosed only through a death certificate (DCO) were excluded from survival analysis.

### Statistical analysis

We estimated net survival up to 5 years after diagnosis for adults diagnosed during 2006–2015, and followed-up to 30 October 2019, by access to care (“easy access to care” versus “poor access to care”), with the non-parametric Pohar-Perme estimator [22]. To estimate net survival, we used life tables of all-cause mortality rates by single year of age for Palermo Province for each calendar year 2006–2018, provided by the Italian National Statistics Institute (ISTAT) [19]. Estimates were age-standardised using International Cancer Survival Standard (ICSS) weights [23] in which age at diagnosis is categorised into five age groups: 15–44, 45–54, 55–64, 65–74, 75–99 years. Differences in survival between the two categories of access to care were assessed using a log-rank-type test [24]. We also examined differences in net survival up to 5 years in primary liver cancer patients by access to care and age (15–64, 65–74 and ≥ 75 years).

To analyse trends in 5-year survival, by ease of access to care, we used the classical cohort approach for patients diagnosed during 2006–2008 and 2009–2011 [25], all of whom had been followed up for at least 5 years, and the period approach for patients diagnosed in 2012–2015 [26].

Statistical analyses were performed by using the IDE software RStudio (version 3.4.1 of 2017-06-30) for R (version 2.1) [27, 28]. Net survival was estimated by using “relsurv” and “periodR” packages [29, 30]. Two-sided statistical significance of the difference between paired survival estimates was set at 0.05.

## Results

Among the 2018 adults with a primary invasive neoplasm of the liver (C22.0), diagnosed during 2006–2015, 1290 (63.9%) occurred in men and 728 (36.1%) in women; 1259 (62.4%) were morphologically verified (including histological or cytological confirmation) and 759 (37.6%) were diagnosed clinically (Table 1, Fig. 1). The median follow-up time for the whole cohort was 1.5 years while the longest follow up was 13.0 years. Only 8 patients (0.4%) were lost to follow-up.

**Table 1:** Demographic characteristics of patients with primary invasive liver neoplasm. Palermo Province Cancer Registry, 2006–2015

Primary invasive liver neoplasm (C22.0)	Total (%)	Sex		Age at diagnosis (years)				
		M (%)	F (%)	15–64 (%)	65–74 (%)	≥75 (%)	Mean	***p***-value
Epithelial liver tumours with defined morphology^a^	**1259** (62.4)	860 (68.3)	399 (31.7)	351 (27.9)	524 (41.6)	384 (30.5)	69	0.0001
Malignant neoplasm with a clinical-instrumental diagnosis^b^	**759** (37.6)	430 (56.7)	329 (43.3)	150 (19.8)	228 (30.0)	381 (50.2)	73	
**Total**	**2018 (100)**	**1290 (63.9)**	**728 (36.1)**	501 (24.8)	752 (37.3)	765 (37.9)		

^a^Epithelial liver tumours: ICD-O-3 codes 8170–8175; 8010; 8020; 8021; 8190; 8246; 8249)

^b^Malignant neoplasms with a clinical-instrumental diagnosis: ICD-O-3 code 8000, behaviour code 3

Epithelial liver tumours with a specified morphology were more frequent in patients aged 65–74 years (41.6%), followed by age-groups ≥75 (30.5%) and 15–64 (27.9%), while malignant neoplasm with a clinical or instrumental diagnosis were more frequent in patients older than 75 years (50.2%), followed by 65–74 (30.0%) and 15–64 (19.8%). Mean age at diagnosis was lower among epithelial liver tumours with defined morphology than those with a clinical or instrumental diagnosis (69 vs.73 years; *p* < 0.0001) (Table 1).

Among 2018 patients, 1259 (62.4%) patients with a morphologically confirmed tumour linked to a PAR and either a HDR or an ADR or both, with a median number of 4 records linked for every single year of survival after diagnosis, while among the remaining 759 patients (37.6%), 585 were identified by at least one HDR or ADR, but no PAR, with a median number of 1 record linked for every single year of survival after diagnosis. Lastly, 174 patients (23%) were linked with a DCO (Fig. 1).

“Easy access to care” was more frequent in patients with a morphologically confirmed liver tumour while patients whose diagnosis was based solely on clinical findings were more frequently characterised by “poor access to care”.

**Fig. 2:**
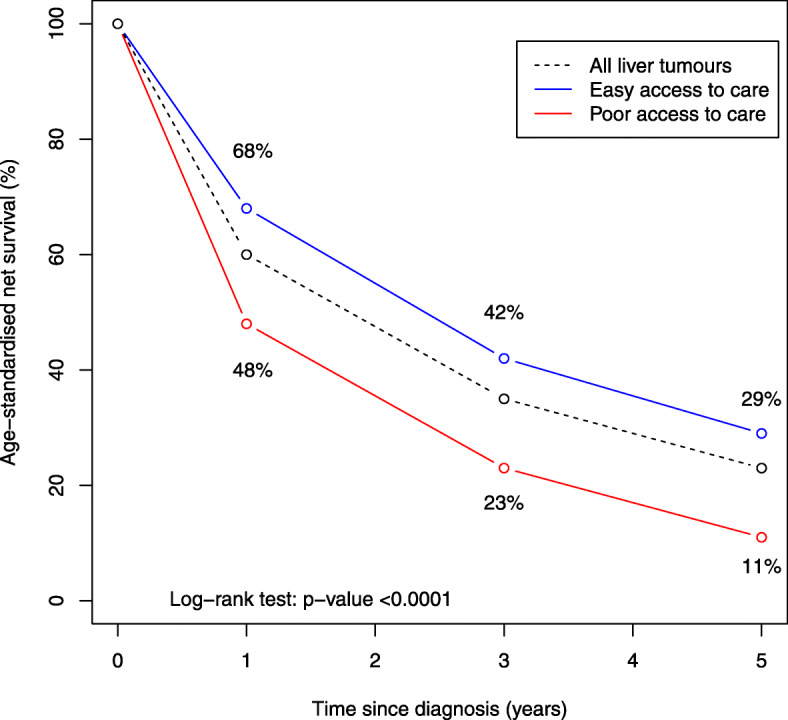
HCC survival up to 5 years, by access to care: Palermo Province Cancer Registry, 2006–2015

During 2006–2015, age-standardised net survival was substantially higher among primary invasive liver cancer patients with “easy access to care” than in those with “poor access to care” (68% vs. 48% at 1 year, 42% vs. 23% at 3 years, 29% vs. 11% at 5 years; *p* < 0.0001; Fig. 2).

**Fig. 3:**
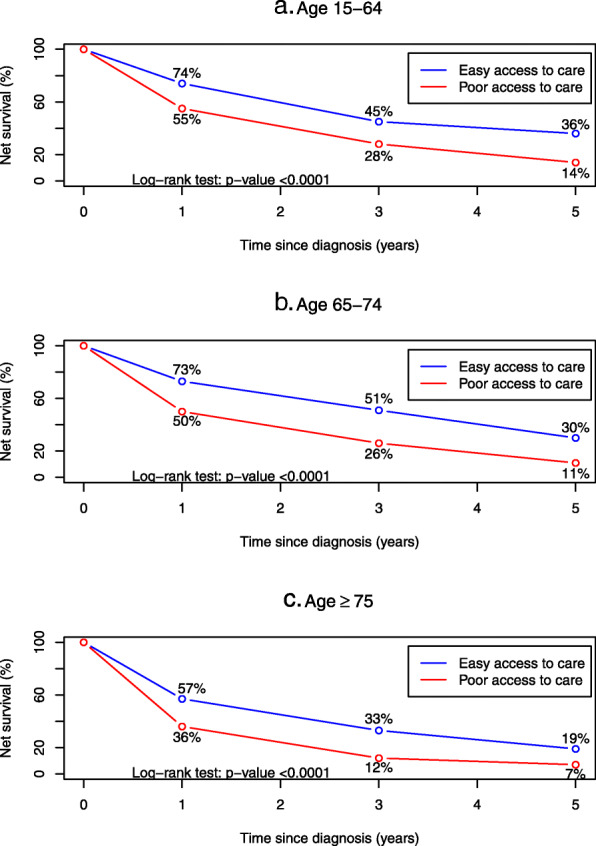
HCC survival up to 5 years, by age and access to care: Palermo Province Cancer Registry, 2006–2015

Survival up to 5 years was higher for patients with “easy access to care” in each age group (*p* = < 0.0001) (Fig. 3).

Survival increased slightly for patients with easier access to care (2006–2008: 26.0, 95%CI 22.7–32.4%; 2009–2011: 30.3%, 24.8–36.0%; 2012–2015: 35.0%, 28.7–41.3%), while it remained relatively stable for patients with poor access to care (2006–2008: 11.8%, 8.2–17.0%; 2009–2011: 11.9%, 8.4–17.7%; 2012–2015: 10.6%, 7.5–19.1%).

## Discussion

Population-based cancer registries play a strategic role in public health, producing continuous valuable information for cancer control strategy and epidemiological surveillance [31], although the informative potential of cancer registries is often underestimated. Surveillance of population-based cancer survival should be used by both national and local health authorities to implement cancer control strategies [32], to prioritise cancer control measures [33], and to assess both the effectiveness [34, 35] and the cost-effectiveness [36] of public health interventions.

This study investigated differences in access to care for primary invasive liver cancer patients, excluding tumours of the intra-hepatic biliary tract, during 10 years of epidemiological surveillance provided by a population-based cancer registry that covers an area in Southern Italy characterised by a high prevalence of HCV and by the presence of 3 highly specialised centres for the treatment HCC patients [37].

We analysed survival up to 5 years for primary invasive liver cancer in two different subgroups of patients for which it was possible to assess the access to care (easy vs. poor) through implementation of a linkage algorithm with the hospital records.

Our findings show that an easy access to care for primary liver cancer was more frequent in patients with a morphologically verified tumour. These patients tend to be younger and to have a higher probability to survive their cancer up to 5 years than those characterised by poor access to healthcare. Moreover, the difference in survival between the two groups of liver cancer patients was present in each age category, supporting the evidence that the better outcome can be mainly attributable to factors related to an easier access to healthcare services [38]. The survival improved overtime in patients with an easier access to care, likely to reflect a progressive improvement in the effectiveness of care services offered to primary liver cancer patients [39].

Survival for liver cancer in Palermo province during 2006–2015 was higher than the average survival in Italy [38]. These results may support the effectiveness of intensive follow-up conducted by a hub-and-spoke network of centres that are specialised in the treatment of hepatitis-related diseases [40] recruiting patients of all ages with tumours at probably earlier stages.

The burden of liver cancer [38], the complexity and cost of its management, which sometimes requires transplantation [41], the peculiarity of the risk factors and their control, have important public health implications, both in terms of primary and secondary prevention policies, and of care that should be accessible and fair all over the world.

We have focused on hepatocellular cancer in consideration of the epidemiologic shifts related to several innovations: the wider use of semi-annual surveillance, that expanded the proportion of tumours qualifying for treatments of curative intent, the improved outcome from loco-regional treatments [17], and the recent access to innovative drugs have driven an improvement of survival for HCC in Italy, particularly after 2009, for patients diagnosed at an early- or intermediate- stage, whether or not the cancer had a viral origin [5, 42–44]. Therefore, the epidemiological scenario of HCC has evolved in terms of both an increasing patient aging and a progressive expansion of non-viral liver cancer cases, namely “metabolic” hepatocellular carcinomas, or cryptogenetic and multi-aetiology cases, together with the ongoing reduction of viral cases [17]. A recent review summarised evidences suggesting that several environmental exposures - in particular to aflatoxin, air pollution, polycyclic aromatic hydrocarbons, asbestos, chimney sweeping occupation, and paints, heavy metals, methyl tertiary-butyl ether, and selenium - may be associated not only with liver cancer but also with non-alcoholic fatty liver disease (NAFLD) [45].

The body of this evidence suggest an increasing need for implementation of prevention strategies to target non-viral risk factors associated with liver diseases and, particularly, with hepatocellular carcinoma. At the same time, beyond the classic epidemiologic surveillance competencies, the role of population-based cancer registries is crucial to produce indicators in support of the economic impact of cancer-related costs analysis and the planning of oncological services [46]. Cancer accounts for an increasing proportion of health care expenditures due to increasing in cancer incidence rates, improvements in diagnostic procedures and treatments, and population aging, the analysis of cancer-related costs, starting from the evidences generated by population-based cancer registries, has been of growing interest for public health planners and policy makers [47]. Studies designed to provide an economic assessment of diagnostic, therapeutic and pathways to care for breast and colorectal cancers [48–50] should perhaps be extended to include liver cancer.

A limitation of this study is that the Barcelona Clinic system routinely used by clinicians to stage HCC is not usually available in population-based cancer registry [51]. A further limitation is related to the lack of information for population-based cancer registries on the aetiology of primary liver tumours (viral, non-viral), which is relevant for clinical progression. Therefore, to better investigate the differences in HCC survival between patients with easy or poor access to care, as documented by our study, we stress the need for a joint effort in HCC staging between clinicians and tumour registries. In line with previous studies, our findings support the importance for the decision-making level to obtain real-world evidence on cancer treatment outcomes from population-based cancer registries, by linking them to regional health care utilisation databases [52, 53]. Moreover, the role of socioeconomic status should also be investigated as it could affect the relationship between survival and access to care [54].

Clinical registries play an import role in identifying priorities to improve access to care [55–57] and clinical outcomes [10, 58, 59] of liver cancer patients with poor access to care. This study emphasises how population-based cancer registries can play a supportive role in meeting the increasing need to prioritise cancer control measures [46].

Future studies using our approach should be performed in order to confirm or refute our findings.

## Conclusions

For a decade (2006–2015), liver cancer survival up to 5 years was higher for patients with easy access to care than for those with poor access. Our experience highlights the importance of the evidence from population-based cancer registries in designing effective policies for cancer control. Our linkage algorithm, applied on a larger scale and for a longer calendar period, could provide valuable evidence to support healthcare decision-making in the context of the evolving epidemiology of hepatocellular carcinoma.

## Data Availability

The data analysed in the current study are available from the corresponding author on reasonable request with permission of Palermo Province Cancer Registry.

## Abbreviations

HCC: Hepatocellular carcinoma
HBV: Hepatitis B virus
HCV: Hepatitis C virus
PPCR: Palermo Province Cancer Registry
ICD-O-3: International Classification of Diseases for Oncology, Third Edition
ENCR: European Network of Cancer Registries
HDR: Hospital Discharge Records
ADR: Ambulatory Discharge Records
PAR: Pathological Anatomy Report
DCO: Death Certificate Only
ISTAT: Italian National Statistics Institute
ICSS: International Cancer Survival Standard
NAFLD: Non-alcoholic fatty liver disease
